# Functional connectivity of the nucleus accumbens predicts clinical course in medication adherent and non-adherent adult ADHD

**DOI:** 10.1038/s41598-025-96780-3

**Published:** 2025-06-04

**Authors:** Ahmed Zaher, Jan Leonards, Andreas Reif, Oliver Grimm

**Affiliations:** 1 Department of Psychiatry, Psychosomatic Medicine and Psychotherapy, University Hospital, Goethe University, Frankfurt am Main, Germany; 2 Fraunhofer Institute for Translational Medicine and Pharmacology ITMP, Theodor-Stern-Kai 7, 60596 Frankfurt am Main, Germany; 3 Klinik für Psychiatrie, Psychosomatik und Psychotherapie, Universitätsklinikum Frankfurt, Goethe-Universität, Heinrich-Hoffmann-Str. 10, 60528 Frankfurt am Main, Germany

**Keywords:** ADHD, Functional connectivity, Nucleus accumbens, Insular cortex, Methylphenidate, Reward system, ADHD, Outcomes research

## Abstract

Attention deficit hyperactivity disorder (ADHD) is a neurodevelopmental disorder that persists into adulthood, contributing to a negative trajectory characterized by worsening symptoms, impaired daily functioning, and reduced quality of life over time. We studied seed-based functional connectivity (FC) as a predictive tool for ADHD’s clinical course. We conducted a longitudinal follow-up of 54 adult ADHD patients who underwent functionale magnetic resonance imaging (fMRI). All patients received stimulant treatment during an initial run-in period. After an average of three years, only subjective responders adhered to treatment (n = 34), whereas non-adherent discontinued (n = 20). We reassessed patients to (1) evaluate the prediction of individual outcome by baseline fMRI and (2) to investigate differences in prediction by baseline fMRI according to long-term treatment vs. discontinuation. We investigated the relationship between nucleus accumbens’ (NAc) FC and symptom development. Reduced FC of the NAc to the default mode network (DMN) associated with higher initial symptom burden, whereas improvement correlated with reduced FC between the NAc and the salience network (SN). In contrast, higher NAc FC to the SN associated with better outcomes in patients receiving long-term treatment, while lower NAc FC to SN was associated with a positive prognosis in non-adherent patients. This work highlights the potential of dopaminergic FC as a prognostic factor in ADHD and the role of the NAc in its prognosis.

## Introduction

Attention deficit hyperactivity disorder (ADHD) stands as a prevalent neurodevelopmental condition characterized by inattentiveness, impulsivity, and hyperactivity. These traits typically emerge in childhood and often persist into adulthood. Globally, the prevalence of ADHD in children is estimated to be between 5 and 7%^1^, while among adults, it is reported to be 3%^2^. Importantly, ADHD is associated with an elevated risk of comorbid conditions across the lifespan of individuals affected by it. In both childhood and adulthood, ADHD patients experience a diverse range of psychiatric comorbidities, including but not limited to, substance use disorders (SUD), depression, as well as diminished quality of life, reduced treatment effectiveness, and even increased mortality when compared to the general population^2–4^.

Despite being established as a neurodevelopmental disorder, the underlying mechanisms of ADHD neuropathology in the involved brain networks remain partially blurred. One pivotal brain network implicated in both ADHD and other psychiatric disorders is the reward network^5^. Numerous studies have consistently highlighted altered neural activity and functional connectivity (FC) patterns in reward circuits among individuals suffering from ADHD when contrasted with neurotypical subjects^5–7^. In particular, the nucleus accumbens (NAc) has been shown to be a central player in the neurobiology of ADHD symptoms^8–10^.

The NAc plays a crucial role in the fronto-striatal circuit, which is implicated in ADHD-related deficits such as impaired delay of gratification and altered reinforcement sensitivity^11^. It is also deeply involved in the dopaminergic system, where psychostimulant medication acts to increase dopamine levels^12^. Lastly, its essential function in reward processing and motivation, which is often impaired in ADHD^13^, means that examining alterations in reward system FC patterns could help identify FC changes that correlate with symptom progression.

Recent studies highlight the importance of individual variation in treatment response and suggest that the relationship between brain changes and clinical improvement is complex. While stimulant medication may induce changes in brain function over time, these effects are more nuanced than previously hypothesized. Van der Pal et al. investigated the effects of methylphenidate on dopamine system development. Using pharmacological MRI, no evidence was found for persistent age-dependent effects of stimulant treatment on dopamine system development, suggesting that previously observed short-term effects may be transient; however the study identified age-dependent associations between medial prefrontal cortex dopamine function and stimulant treatment, though these were not correlated with ADHD symptom severity^14^.

A comprehensive voxel-wise mega-analysis^15^ across multiple cohorts demonstrated widespread subcortico-cortical dysconnectivity in ADHD, while longitudinal studies^16^ using the ABCD dataset have revealed that stimulant exposure affects striatal connectivity networks over time. Furthermore, cortical alterations have been associated with differential responses to methylphenidate treatment in adults with ADHD^17^, highlighting the potential for neuroimaging biomarkers to predict treatment outcomes^18^.

**Fig. 1:**
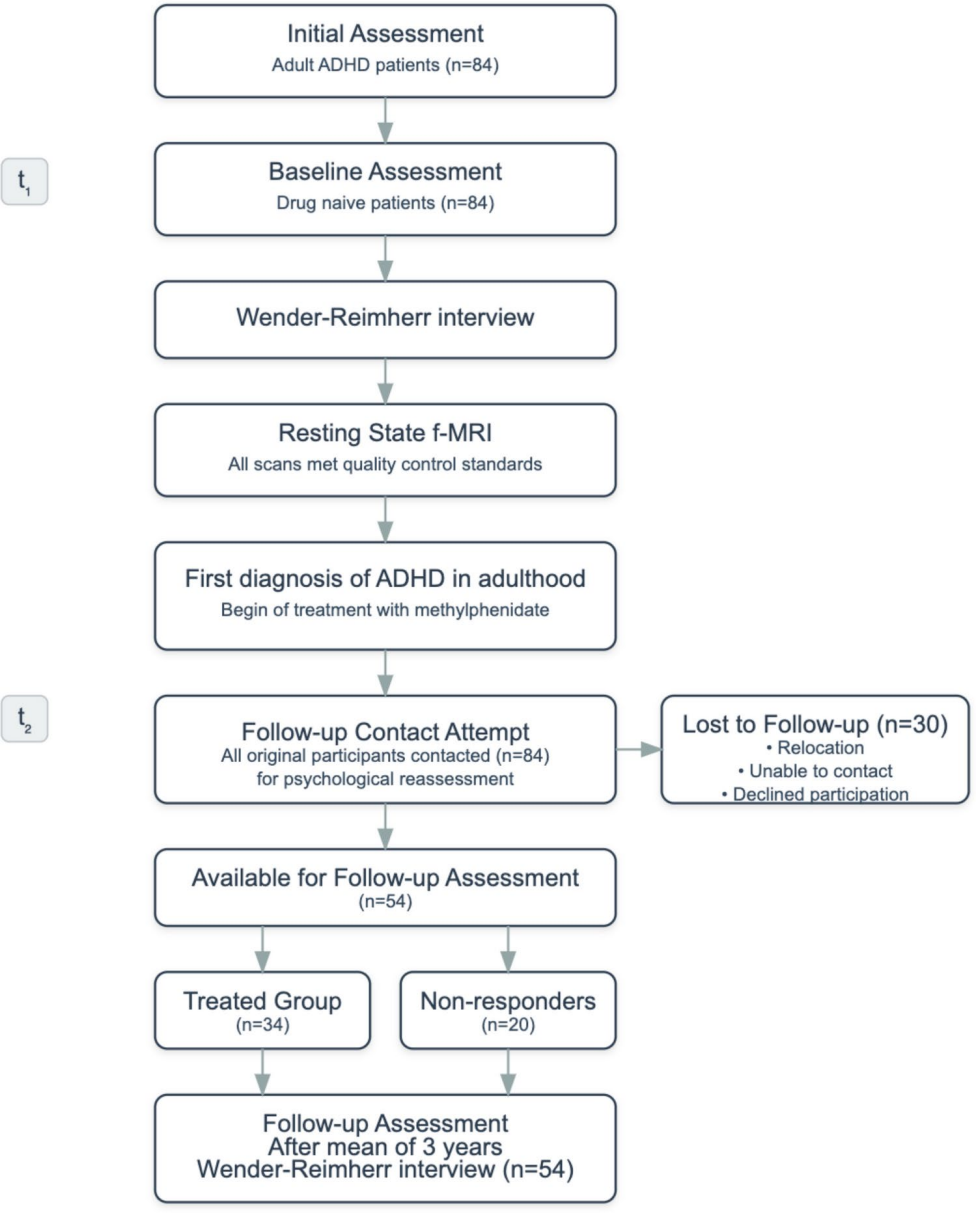
Study workflow.

Our study’s hypotheses were twofold. First, we hypothesized that resting-state FC patterns of the NAc at baseline (*t₁*) would predict the outcome of ADHD symptoms in adults, when those are re-evaluated after years of first diagnosis (*t₂*). Specifically, we expected that connectivity between NAc and regions involved in executive control and reward processing would predict clinical outcomes, based on previous evidence showing disrupted reward circuit connectivity in ADHD. A clinical follow-up approximately three years later (*t₂*) was conducted, after conducting baseline f-MRI scans and comprehensive clinical evaluation at the point of diagnosis (*t₁*). Second, we hypothesized that baseline NAc seed-connectivity patterns would correlate with dopaminergic neurotransmitter maps, particularly dopamine transporter (DAT) density, given methylphenidate’s mechanism of action as a DAT inhibitor. This hypothesis was founded on the well-established role of dopaminergic dysfunction in ADHD pathophysiology and the known effects of stimulant medications on dopamine signaling. To test these hypotheses, we measured ADHD symptoms using the Wender-Reimherr-Interview (WRI) at both timepoints, calculated symptom changes over time (*∆* WRI: WRI *t₂*—WRI *t₁*), and correlated these clinical outcomes with baseline resting-state fMRI brain scans. We examined correlations between NAc connectivity patterns and established neurotransmitter receptor and transporter maps to validate our findings within the context of dopaminergic transmission. Figure 1 shows the workflow of our study.

## Methods

### Participants

The study includes n = 54 physically healthy ADHD patients who received their diagnosis in adulthood at *t*_*1*_ (for demographics see Table 1). Recruitment took place at the University Hospital, Goethe University Frankfurt am Main. Inclusion criteria were (1) age between 18 and 50 years; (2) sufficient understanding of the German language and, (3) established ADHD diagnosis on the basis of the DSM-IV criteria. Exclusion criteria were other mental illnesses (apart from ADHD, depression and SUD), serious acute or chronic physical diseases, pregnancy, as well as exclusion criteria of the MRI examination.

**Table 1:** Demographics (participants, n = 54); M: mean; SD: standard deviation; MA: medication adherent group; MNA: medication non adherent group.

	MA *t*_*2*_ (n = 34)	MNA *t*_*2*_ (n = 20)	statistics	*p*	SD	Range: Min/Max
Female male, n (%)	14 (25.9) 19 (35.2)	9 (16.7) 12 (22.2)	χ^2^ < 0.01	0.975	n.a	n.a
Age, M in years	30.9	30.1	F = 0.113	0.738	8.32	18/50
WRI *t*_*1*_, M	14	15	F = 1.23	0.294	4.16	6/22
WRI *t*_*2*_, M	3.3	10	F = 36.06	0.001	5.55	0/21
∆ WRI (*t*_*2*_*-t*_*1*_), M	− 10.6	− 5.10	F = 12.2	0.001	5.85	− 21/0

The approval to conduct the study was given by the local ethics commission (Faculty of Medicine, University Hospital, Goethe University, Frankfurt am Main) and was subject to the Declaration of Helsinki of the “World Medical Association: Ethical Principles for Medical Research Involving Human Subjects” and the “Guidelines for Good Clinical Practices (GCP)”. In addition, the study was registered as a clinical trial in the German study registry under the ID: DRKS00011209. Written informed consent was obtained from each volunteer before the start of the study.

Initially at *t*_*1*_ a total of 84 adult ADHD patients from our outpatient clinic underwent baseline MRI scanning (*t₁*). The diagnosis was established using the Wender-Reimherr-Interview (WRI) that measures the severity of ADHD symptoms. After initial diagnosis patients received their MRI scanning. After these scans all subjects were prescribed methylphenidate as part of their routine clinical care. After a mean period of 6 months during clinical care a group of 30 subjects did not respond to medication and stopped the medication. All participants received methylphenidate as part of routine clinical care, not within a clinical trial protocol. The prescription of methylphenidate took place approximately 1–3 weeks after the fMRI measurement. Initial dosing and subsequent adjustments followed standard clinical practice guidelines^19^ for adult ADHD. Starting doses were typically 10–20 mg/day of immediate-release/extended-release methylphenidate with subsequent titration based on individual response and tolerability. Final maintenance doses ranged from 20 to 80 mg/day in accordance with standard therapeutic ranges for adult ADHD. Treatment response was determined through clinical assessment based on patients’ self-reported improvement in their symptoms during a six month follow-up consultations after the initiation of medication. At t₂, 30 patients were lost to follow-up due to various reasons including relocation, inability to contact partients, and declining further participation. The remaining 54 participants completed the follow-up psychological assessment (t₂) and comprised our final analysis sample. At follow-up, this sample naturally divided into two groups based on medication adherence: 34 participants who continued methylphenidate treatment; medication adherent group (MA) and 20 who had discontinued treatment due to insufficient response; medication non-adherent group (MNA). Statistical comparisons revealed no significant differences between these groups in age (MA: M = 30.9 years; MNA: M = 30.1 years; F = 0.113, *p* = 0.738) or gender distribution (MA: 14 female, 19 male; MNA: 9 female, 12 male; χ^2^ < 0.01, *p* = 0.975). However, there was a significant difference in symptom change (Δ WRI) between groups (MA: M = − 10.6; MNA: M = − 5.10; F = 12.2, *p* = 0.001), indicating greater symptom improvement in the MA group.

The mean interval between *t*_*1*_ and *t*_*2*_ assessments was 3.24 years (SD = 1.00; 95% CI [2.97, 3.51]). Initial *t*_*1*_ assessments were conducted between 2016 and 2019, while all follow-up assessments *t*_*2*_ were completed in 2021. The minimum interval between *t*_*1*_ and *t*_*2*_ was 2 years and the maximum was 5 years, with a median interval of 3.00 years.

### The Wender-Reimherr-Interview (WRI)

A trained and results-blinded registered psychiatrist conducted the German version of the Wender-Reimherr-Interview (WRI)^20^ to assess ADHD-specific symptoms in study participants.

The WRI is a structured psychopathological interview that assesses the severity of ADHD symptoms in seven categories including: attention difficulties, hyperactivity, temperament, affective lability, emotional reaction to stress, disorganization, and impulsivity. Each category is assigned a score depending on the severity of the symptom. An overall global score is calculated based on the seven categories. We used the individual patients’ global scale score in our statistical analysis.

We calculated the change of scores over time (Δ WRI: *t*_*2*_*−t*_*1*_) by subtracting the global WRI-score of the first assessment (WRI *t*_*1*_) from that of the second assessment (WRI *t*_*2*_).

## Data analysis: fMRI

### fMRI acquisition

For the fMRI measurements in our study, we used a 3 Tesla full-body MR scanner (Siemens Magnetom Trio syngo MR A35) at the Brain Imaging Center in Frankfurt am Main. An eight-channel head coil was also utilized for data acquisition. We acquired a t1-weighted sequence (MPRAGE) with a duration of 4:28 min, as well as a gradient echo sequence for functional imaging data, which lasted 8:01 min. The sequence parameters for the MPRAGE sequence were as follows: repetition time (TR) = 1900 ms, echo time (TE) = 3.04 ms, TI = 900 ms, flip angle = 9, field of view (FoV) = 256 × 256 mm, and voxel size = 1 × 1 × 1 mm. For the EPI sequence, the parameters were as follows: TR = 1800 ms, TE = 30 ms, flip angle = 90, FoV = 192 × 192 mm, 28 layers with 4 mm thickness, and voxel size = 3 × 3 × 4 mm. To minimise head movement during the MRI scans, we used foam pads for the study participants.

### Pre-processing of fMRI Data

Results included in this manuscript came from analyses performed using CONN^21^ (RRID:SCR_009550) release (12) CONN—functional connectivity toolbox v18.b^22^ and SPM 12^23^ (RRID:SCR_007037) release 12.7771.

#### Preprocessing

Potential outlier scans were identified using ART^24^ as acquisitions with framewise displacement above 0.9 mm or global BOLD signal changes above 5 standard deviations^25,26^. A reference BOLD image was computed for each subject by averaging all scans excluding outliers.

#### Denoising

In addition, functional data were denoised using a standard denoising pipeline^27^ including the regression of potential confounding effects characterized by white matter timeseries (5 CompCor noise components), CSF timeseries (5 CompCor noise components), session and task effects as well as their first order derivatives (6 factors), outlier scans (below 133 factors)^25^, motion parameters and their first order derivatives (12 factors)^28^, and linear trends (2 factors) within each functional run, followed by bandpass frequency filtering of the BOLD timeseries^29^ between 0.008 and 0.09 Hz. CompCor^30,31^ noise components within white matter and CSF were estimated by computing the average BOLD signal as well as the largest principal components orthogonal to the BOLD average, motion parameters, and outlier scans within each subject’s eroded segmentation masks.

#### Region of interest analysis

In our study, we used a seed-based connectivity analysis based on subcortical region-of-interest (ROI) seeds. The NAc was selected from brain regions that are central to the mesolimbic brain reward systems, as previously defined in the OTI Atlas^32^. This atlas was constructed using high-spatial resolution t1- and t2-weighted structural images, with tissue boundaries used to delineate subcortical nuclei, which were then combined to form a probabilistic atlas. From this atlas, we selected the NAc as our seed ROI for the connectivity analysis.

### Conn toolbox: first level analysis: seed to voxel models

Seed-based connectivity maps (SBC) were estimated characterizing the patterns of functional connectivity. Functional connectivity strength was represented by Fisher-transformed semipartial correlation coefficients from a weighted general linear model (weighted-GLM^27^), defined separately for each target area, modeling the association between all seeds simultaneously and each individual target area BOLD signal timeseries. Individual scans were weighted by a boxcar signal characterizing each individual task or experimental condition convolved with an SPM canonical hemodynamic response function and rectified. The residual BOLD-time course was extracted from the seed ROI (NAc) and correlated with other voxels in the brain to calculate the first-level correlation maps. We then transformed the first-level correlation coefficient maps into a normally distributed z-score.

### Conn toolbox group statistics

#### Statistical framework

Group-level analyses were performed using a general linear Model (GLM^27^). For each individual voxel a separate GLM was estimated, with first-level connectivity measures at this voxel as dependent variables (one independent sample per subject and one measurement per task or experimental condition, if applicable), and groups or other subject-level identifiers as independent variables. Voxel-level hypotheses were evaluated using multivariate parametric statistics with random-effects across subjects and sample covariance estimation across multiple measurements.

#### Multiple comparison corrections

For correction of multiple testing during second-level statistics we used cluster-wise whole-brain analysis which uses a combination of an uncorrected *p* < 0.001 height threshold to initially define clusters of interest from the original statistical parametric maps, and an FDR-corrected *p* < 0.05 cluster-level threshold to select the significant clusters among the resulting clusters.

Inferences were performed at the level of individual clusters (groups of contiguous voxels). Cluster-level inferences were based on parametric statistics from Gaussian Random Field theory^27,33^. Results were thresholded using a combination of a cluster-forming *p* < 0.001 voxel-level threshold, and a familywise corrected *p*-FDR < 0.05 cluster-size threshold^34^.

By using a more stringent threshold of *p* < 0.001 for FDR cluster analysis, we aimed to mitigate the issue of inflated false positive rates. While this threshold may result in a lower number of false positives compared to less liberal thresholds, it also has the potential to increase the number of false negatives (i.e., failing to detect true effects). However, given the findings of Eklund et al.^35^ it is important to prioritize controlling the false positive rate to ensure the reliability and validity of fMRI results.

#### CONN toolbox group statistics

First, we performed a regression test to calculate a possible relationship between the initial symptom load (WRI t₁) and the FC of NAc. Second, we performed a regression analysis examining the relationship between FC and symptom change over time (Δ WRI) across all patients (n = 54), with age and sex included as covariates to account for demographic variability.

To then explore whether the neural basis of connectivity patterns differed between MA and MNA patients at t₂, we conducted a separate one-way ANCOVA to investigate the effect of group (MA vs. MNA) on the FC within the reward system using NAc as the seed. We used a regression model that included the following regressors: age, sex, medication status (binary: 0 or 1), MNA status (binary: 0 or 1), *Δ* WRI of MA, and *Δ* WRI of MNA. This allowed us to model the relationship between FC and *Δ* WRI separately for MA and MNA. We applied a contrast vector [0, 0, 0, 0, 1, − 1], which compares the regression slopes of *Δ* WRI for MA vs. MNA, effectively testing for an interaction between medication adherence status and *Δ* WRI in predicting FC. This approach is consistent with established practices in neuroimaging data analysis, where contrasts are used to test for interactions between groups and continuous variables^21,36^. By using the contrast [0, 0, 0, 0, 1, − 1], we effectively tested for an interaction between responder status and Δ WRI, even though the interaction term was not explicitly included in the model formula.

### Data analysis: spatial correlations with neurotransmitter maps

To investigate the biological underpinnings of NAc seed connectivity and validate its interpretation within the context of dopaminergic signaling, we examined the spatial correspondence between accumbens-connectivity maps and neurotransmitter receptor maps derived from PET imaging. Given the established role of dopamine in ADHD and methylphenidate’s function as a dopamine transporter inhibitor^37^. We hypothesized that accumbens connectivity maps would correlate with D1- and D2-receptor density and dopamine transporter availability. We used the 5-HT1a-receptor (serotonin 5-hydroxytryptamine receptor subtype 1a) as a control condition, expecting no significant correlation. We utilized published PET/SPECT maps for the following receptors: 5-HT1a, D1 (dopamine D1), D2 (dopamine D2), DAT (dopamine transporter), and F-DOPA (dopamine synthesis capacity). The AAL-brain atlas was employed for brain region parcellation. We then calculated correlations between all PET/SPECT maps and accumbens-seed connectivity maps across all subjects (n = 54). In Figure 5A, the results of these single-subject maps are combined into a one-sample t-test, highlighting the distribution of NAc-connectivity. For this analysis, we employed the JuSpace-toolbox, a MATLAB-based software package designed for spatial correlation analyses between neuroimaging data and receptor density maps^38^. To assess the statistical significance of these correlations, the toolbox generates null-maps using a permutation-based approach, which randomly shuffles the original neuroimaging data while preserving spatial autocorrelation, creating a null distribution for comparison.

## Results

### ADHD symptom severity

In order to measure the ADHD symptoms load, the global score of the WRI was calculated at the time of diagnosis and at the time of follow up. WRI *t*_*1*_ showed (M = 14.5, SD = 4.16, Min/Max = 6/22), WRI *t*_*2*_ showed (M = 6.06, SD = 5.55, Min/Max = 0/21). The *Δ* WRI showed (M = − 8.46, SD = 5.86, Min/Max = − 21/0). The mean percentage change in WRI scores was: -58.53%. (Supplementary Figure S1 shows a repeated measure raindrop chart of the WRI scores at *t*_*1*_ and *t*_*2*_).

### ADHD symptoms and functional connectivity of the NAc

#### ADHD symptoms at baseline and FC in the total sample

**Fig. 2:**
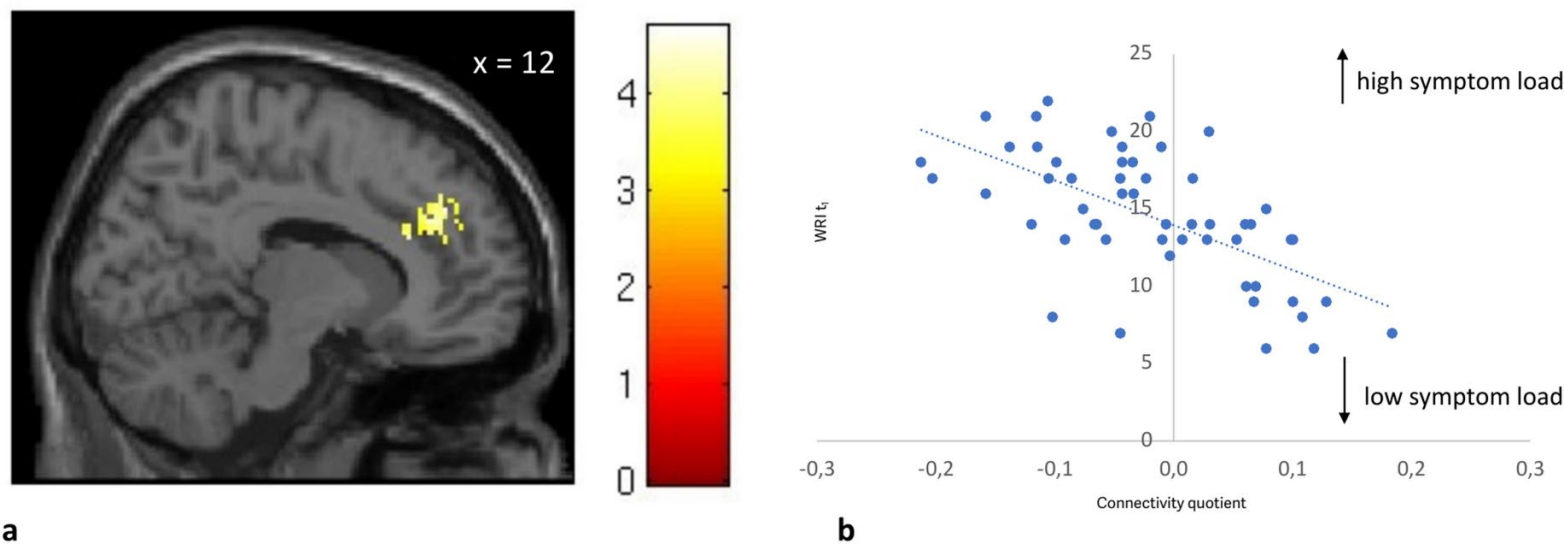
Correlation between global score WRI *t*_1_ and the FC-quotient between NAc and the paracingulate Gyrus right in all subjects. In 2a Statistical *t*-map showing the significant cluster of the connectivity analysis: the paracingulate gyrus right and its localization is demonstrated at the MNI *x* = 12 plane. In 2b a scatterplot of individual FC-Quotient against global score WRI *t*_1_ of each subject is shown. FC: Functional connectivity; NAc: Nucleus accumbens; WRI: Wender-Reimherr-Interview. *Supplementary Figure S2 shows the glass brain image of the above shown cluster.

A significant correlation was observed between the severity of symptoms at the time of diagnosis (WRI *t*_*1*_) and the FC between the NAc and the right paracingulate gyrus (r = − 0.618, *p* < 0.001), where a lower connectivity between the NAc and the paracingulate gyrus correlated with higher severity of ADHD symptoms in adulthood. (Cluster k = 93; MNI-Coordinates (x, y, z): 12, 42, 32; pFDR = 0.01; t = − 5.64) as shown in Fig. 2.

#### Change in ADHD symptoms and FC in the total sample

**Fig. 3:**
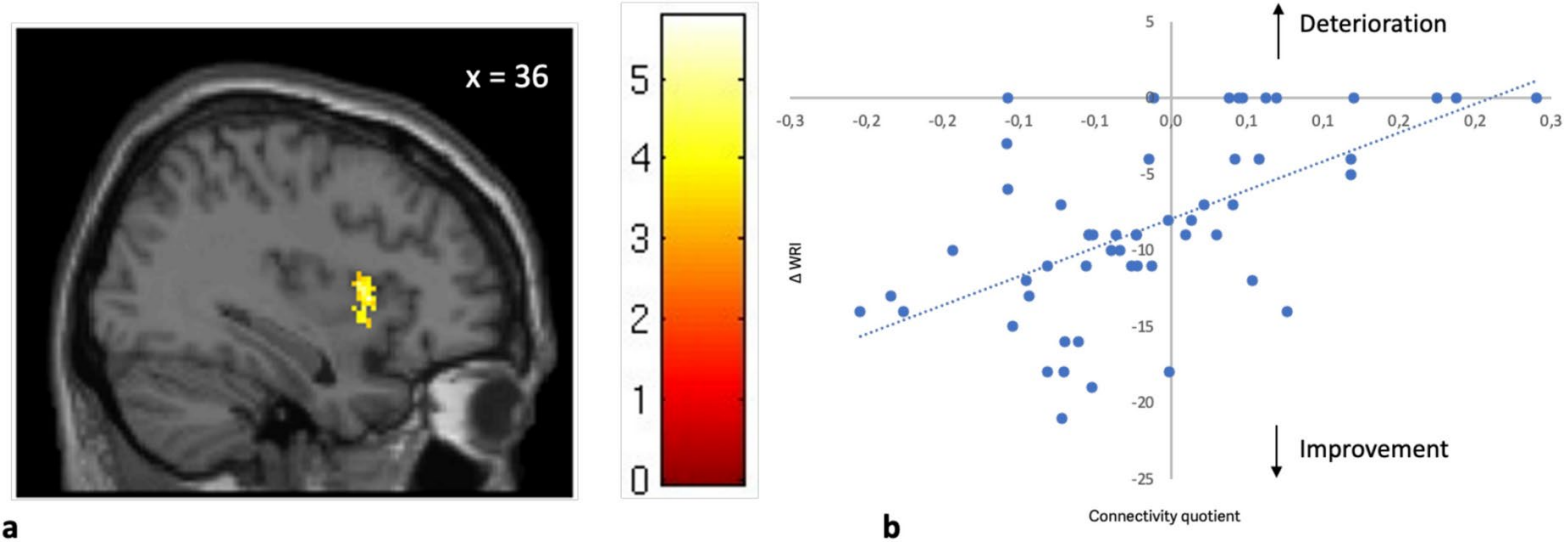
Correlation between ∆ WRI (*t*_2_-*t*_1_) and the FC-quotient between the NAc and the anterior insula right in all subjects. In 3a Statistical t-map showing the significant cluster of the connectivity analysis: the anterior insula right and its localization is demonstrated MNI *x* = 36 plane. In 3b a scatterplot of individual FC-quotient against ∆ WRI each subject is shown. NB: Arrow direction explains the development of symptoms over time. FC: Functional connectivity, NAc: Nucleus accumbens, WRI: Wender-Reimherr-Interview. *Supplementary Figure S2 shows the glass brain image of the shown cluster.

A significant correlation was observed between the long-term change of the global score: *Δ* WRI (*t*_*2*_*-t*_*1*_) and the FC between the NAc and the insular cortex (r = 0.583, *p* < 0.001), where a lower connectivity between the NAc and the right insular cortex predicted a better outcome of the ADHD symptoms. (Cluster k = 101; MNI-Coordinates (x,y,z) : 36, 12, 4; pFDR = 0.01; t = 5.33) as shown in Fig. 3.

#### ADHD symptom severity in continued versus discontinued treatment

**Fig. 4:**
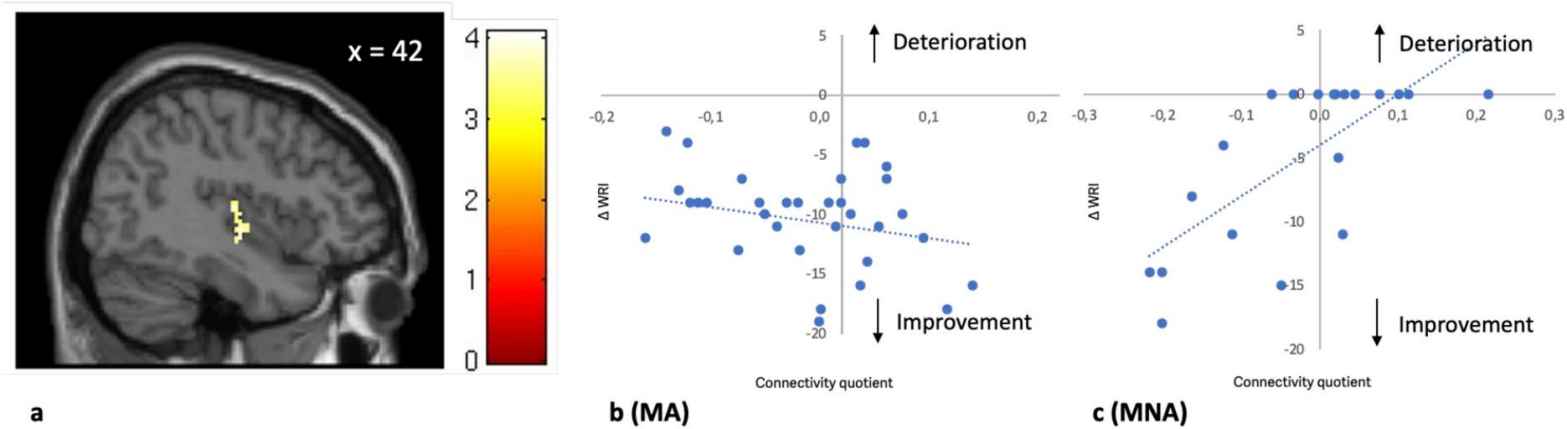
Correlation between ∆ WRI (*t*_2_-*t*_1_) and the FC-quotient between NAc and the posterior insula in MA vs. MNA. In 4a Statistical t-map showing the significant cluster of the connectivity analysis: the posterior insula right and its localization is demonstrated MNI *x* = 42 plane. In 4b a scatterplot of individual FC-quotient of MA patients against ∆ WRI of those subjects is shown. In 4c a scatterplot of individual FC-quotient of MNA patients against ∆ WRI of those subjects is shown. NB: Arrow direction explains the development of symptoms over time. FC: Functional connectivity, NAc: Nucleus accumbens, WRI: Wender-Reimherr-Interview. *Supplementary Figure S2 shows the glass brain image of the shown cluster.

Next, we compared the correlation of FC to *Δ* WRI in those receiving stimulants and those not at *t*_*2*_. In those receiving treatment, a higher FC between the NAc and the insular cortex predicted a better overall long-term outcome. Meanwhile we observed that in those not receiving medication at *t*_*2*_ higher FC predicted a worse outcome, (Cluster k = 91; MNI-Coordinates (x,y,z): 42, − 12, − 2; pFDR = 0.04; t = 4.88) as shown in Fig. 4.

### Regression analysis

In a further step to examine the validity of the conducted CONN toolbox statistical analysis. We extracted the individual connectivity quotients of each patient and re-appraised the statistical relations between the connectivity quotients and the individuals scale scores.

A linear regression analysis was conducted to examine the effect of functional connectivity on WRI scores at *t*_*1*_. The regression model was significant, F(1.52) = 32.207, *p* < 0.001, with an R = 0.618, R^2^ = 0.382, adjusted R^2^ = 0.371, indicating that functional connectivity explains 38% of the variance in clinical scale scores at *t*_*1*_. Functional connectivity at *t*_*1*_ significantly predicted WRI scores at *t*_*1*_, B = − 29.030, SE = 5.115, β = − 0.618, t(98) = − 5.675, *p* < 0.001.

A linear regression analysis was conducted to examine the effect of the functional connectivity on ∆ WRI (*t*_*2*_*-t*_*1*_). The regression model was significant, F(1.52) = 26.707, *p* < 0.001, with an R = 0.583, R^2^ = 0.339, adjusted R^2^ = 0.327, indicating that functional connectivity explains 33.9% of the variance in clinical scale score difference. Functional connectivity at *t*_*1*_ significantly predicted the clinical scale score change from *t*_*1*_ to *t*_*2*_, B = 37.694, SE = 7.294, β = 0.58, t(98) = 5.168, *p* < 0.001.

An ANCOVA was conducted to examine the effect of medication status on FC while controlling for WRI scores. The overall model was not statistically significant, F(2,51) = 2.579, *p* = 0.086, explaining 9.2% of the variance in FC (R^2^ = 0.092). Medication status did not have a significant effect on FC after controlling for ∆ WRI (*t*_*2*_*-t*_*1*_), F(1,51) = 0.451, *p* = 0.505, partial η^2^ = 0.009. However, WRI scores significantly predicted FC, F(1,51) = 5.011, *p* = 0.030, partial η^2^ = 0.089, indicating that higher ∆ WRI (*t*_*2*_*−t*_*1*_) scores were associated with changes in FC.

### Functional connectivity of the NAc and the expression of dopamine receptors

**Fig. 5:**
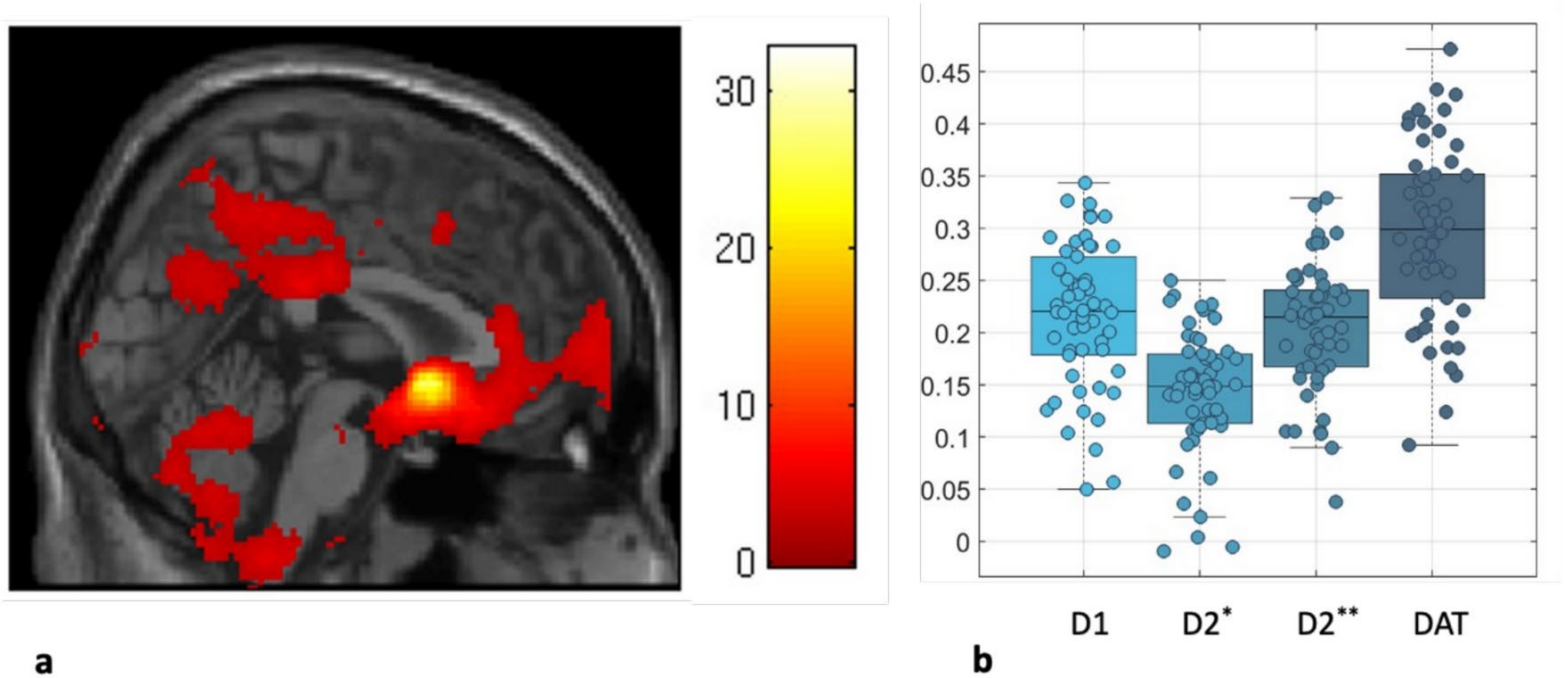
Correlation between NAc connectivity and the densities of D1, D2 and DAT. In 5a a Statistical *t*-map showing significant clusters of the connectivity analysis: encompassing the precuneus cortex, the frontal pole right, the posterior cingulate gyrus. For illustrative purposes a *p* < 0.01 was used for this analysis. In 5b a box plot displays the Fisher’s z-transformed Pearson correlation coefficients for D1, D2, and DAT in each subject. *raclopride ligand, **fallypride ligand.

The correlation analysis revealed that NAc connectivity was positively associated with both D1 and D2 receptor densities as shown in Fig. 5. Specifically, D1 receptor density exhibited moderate positive correlations with accumbens connectivity, suggesting that regions with stronger connectivity to the NAc are also characterized by higher D1 receptor densities. Similarly, D2 receptors, assessed using both raclopride and fallypride, also showed moderate positive correlations, indicating that D2 receptor-rich regions are functionally connected to the NAc. These findings are in line with the dopaminergic modulation role of D2 receptors in ADHD.

Notably, the strongest correlation was observed with DAT availability, where the NAc connectivity exhibited a robust positive relationship with dopamine transporter density. This result underscores the critical role of dopamine reuptake in regulating accumbens connectivity and further highlights the significance of DAT in ADHD pathology. Given methylphenidate’s mechanism of action as a DAT inhibitor, our findings provide a neurobiological explanation for its therapeutic effects in ADHD by modulating NAc connectivity.

### Movement-related effects

We extracted mean-motion and max-motion values from the MA vs. MNA (n = 54) and performed independent samples t-tests. MA and MNA patients did not show significant motion changes (*p* > 0.05). We did not detect any differences between the two groups in mean-motion (*p* = 0.25) or max-motion (*p* = 0.55) (see Table 2).

**Table 2:** Movement-related effects in all MA and MNA subjects (participants, n = 54; MA, n = 34; MNA, n = 20); FD: framewise displacement; SD: standard deviation.

	MA *t*_*1*_	MNA *t*_*1*_
Mean-motion FD; SD	0.25; 0.17	0.20; 0.20
Mean of max-motion; SD	1.59; 0.06	1.41; 0.98

## Discussion

Our study found that changes in brain connectivity between the NAc and the paracingulate cortex (PCC) and insular cortex (IC) predicted symptom improvement between the two time points *t*_*1*_ and *t*_*2*_. The direction of FC patterns differs depending on whether medication is continued or discontinued due to treatment failure.

It’s crucial to emphasize that our study did not directly compare the FC at *t*_*1*_ and *t*_*2*_. Instead, we aimed to use resting state FC patterns observed at *t*_*1*_ as predictors for symptom development and treatment response at *t*_*2*_.

This predictive approach allows us to explore whether initial brain connectivity patterns can forecast the trajectory of ADHD symptoms and treatment outcomes over time. By focusing on FC as a predictor, we seeked to identify potential neurobiological markers that could inform early intervention strategies and personalized treatment plans. This distinction is important for interpreting our results, as we are not tracking changes in FC, but rather investigating how baseline FC relates to future clinical outcomes.

First, analysis showed that a higher symptom load at *t*_*1*_ correlated with decreased FC between the NAc and the right paracingulate gyrus: an important region of the default mode network of the brain (DMN), reflecting previous reports that ADHD patients exhibit hypo-connectivity between the reward system and the DMN^39–41^. Improvement in symptoms over time was associated with decreased FC between the NAc and the right anterior insular cortex (aIC) of the salience network (SN).

Second, when considering the medication status of subjects at *t*_*2*_, a significant correlation emerged between receiving stimulant medication and improvement in global symptom scores. Dividing our subjects into two groups based on their medication status at *t*_*2*_, we found in the MA group, a higher FC between the NAc and the right posterior insular cortex (pIC) correlated significantly with improvement under medication. Conversely, improvement in the MNA group correlated with lower FC between the NAc and the right pIC.

These findings have to be interpreted from the background of previously described alterations in the reward system in ADHD patients^39,42,43^: The reward circuitry in humans, including the ventral tegmental area (VTA), the ventral striatum, and dopaminergic projections to the NAc, forms a crucial part of the brain’s reward system^44^. In the pathophysiology of ADHD symptomatology, the brain reward circuit is considered to be one of the major brain hubs involved where changes in the functional connectivity of the NAc and the DMN have been implicated in the development of ADHD^45^. In particular, deficits within the brain’s reward system and alterations in the connectivity of the NAc are observed in ADHD patients^46^. Children suffering from ADHD have been recently shown to have altered brain network developmental trajectories between cortico-limbic regions and between visual and higher-order cognitive networks^47^.

Our subjects displayed a significant correlation between higher ADHD symptom load and the FC of the NAc to the right paracingulate gyrus. Hypo-connectivity between these regions correlated positively with symptom load. The paracingulate gyrus, part of the cingulate cortex in the DMN, is active during rest and self-referential mental activities^48^. Prior studies have established structural connections between the NAc and the DMN, indicating a potential influence on ADHD symptomatology^45^. The DMN is active during rest, involved in self-referential thoughts, and mind-wandering. Altered DMN activity could interfere with an individual’s ability to sustain attention. Interactions between the DMN and other brain networks, such as the SN, involve distinct topological structures that support reward processing and emotion regulation^49^. These findings are consistent with a recent mega-analysis by Norman et al.^15^ that demonstrated widespread subcortico-cortical dysconnectivity in ADHD, particularly affecting reward and attention networks.

Our study shows that patterns of FC between these key areas can play a role in predicting the course of ADHD-symptoms. Generally decreased FC between the DMN and the NAc correlated with high symptom load.

In ADHD individuals, reduced FC between the DMN and the reward system suggests disruptions in cognitive control processes related to reward processing. The dorsal anterior insula (dAI), responsible for cognitive control, appears to be implicated in these disruptions.

The decreased FC between the reward system and the DMN suggests that individuals with higher ADHD symptom load experience disruptions in their ability to effectively control cognitive processes related to reward processing.

Apart from altered connections to the DMN and dACC, ADHD patients’ symptom improvement over time, as measured by the change in WRI, was significantly linked to reduced functional connectivity between the NAc and the anterior insular cortex (aIC).

The insular cortex is a complex, highly connected cortical hub that plays a critical role in interoception, self-awareness, and emotional regulation^50^, and is key in managing attention through its interactions with the fronto-parietal network (FPN) and the DMN^51,52^. As a central structure in the SN, it is pivotal in transitioning between the DMN and other networks, assessing the relevance of sensory information and mental events^53,54^. Notably, adults with persistent ADHD symptoms exhibit reduced insular density, especially thinning of the left insular grey matter in combined subtype ADHD^55^. The observed FC patterns and their relationship to ADHD symptom changes can be partly explained by the ventral anterior insula (vAI) subdivision, linked to affective processes. Increased FC between the reward system and the anterior insula in individuals with unimproved or worsening symptoms may suggest amplified affective responses to rewards, potentially exacerbating symptoms. Conversely, symptom improvement associated with reduced FC might indicate normalized affective responses. Upon examining medication status, we found a significant correlation between receiving stimulant treatment at the second time point (*t*_*2*_) and symptom improvement, as indicated by the WRI at *t*_*2*_. This suggests a relationship between symptom amelioration and stimulant medication. MA patients at *t*_*2*_ exhibited a pattern where increased FC between the NAc and the pIC corresponded with WRI-scale improvement. In contrast, MNA, who did not improve and were not on medication at *t*_*2*_, showed an opposite pattern, where increased FC was linked with symptom deterioration. This pattern suggests that in ADHD individuals, the pIC might emit stronger, possibly dysregulated interoceptive signals, contributing to symptom complexity.

Medication, by influencing the connectivity between the pIC and the reward system, may help regulate these interoceptive signals (or urges) and mitigate impulsive responses. This interpretation aligns with the concept that the insula’s original role in urge processing was evolutionarily geared toward satisfying primary biological needs. Stimulants like methylphenidate (MPH) or amphetamines can modulate connectivity patterns in the brain, affecting neural networks associated with attention and impulse control. The differences in connectivity changes between the MA and MNA groups may be due to the mechanisms of stimulant treatment. Wong et al. showed that stimulant drugs had an effect on the FC of brain FPN^56^. Picon et al. found that ADHD patients exhibited increased connectivity within the DMN, specifically between the Posterior Cingulate Cortex (PCC) and Lateral Parietal Cortex (LPC) after methylphenidate treatment^57^. However, this likely reflects the prediction independent of the medication continuation status.

Although at a group level MPH has been proven to be effective in ameliorating symptoms, there is individual variability in response, which affects clinical outcomes as shown by the two groups. Adherence to medication in ADHD treatment is influenced by multiple factors, including pharmacological tolerability, adverse effects, healthcare accessibility, and patient preferences, which are not necessarily related to therapeutic efficacy. While patients may discontinue medication despite symptomatic improvement due to intolerable side effects, the perceived therapeutic benefit, particularly with rapid-acting psychostimulants used in ADHD treatment, remains a crucial factor in medication continuation. This is supported by evidence showing that higher MPH doses are associated with improved treatment response and subsequent medication adherence^58^. Recent epidemiological research has highlighted the significance of patient-specific factors in medication adherence patterns. Gémes et al.^59^ demonstrated that sociodemographic variables and psychiatric comorbidities significantly influence treatment continuation in adult ADHD. Their findings revealed that patients with affective comorbidities showed better medication adherence, suggesting a potentially enhanced therapeutic response in this subgroup. This observation may help explain why some subjects of the MNA group, despite medication discontinuation, demonstrated improved scores on the Wender-Reimherr Interview (WRI). Their symptom improvement might be attributed to alternative factors such as spontaneous symptom remission or non-pharmacological interventions.

While medication non-adherence in adult ADHD may partially reflect insufficient therapeutic efficacy, patient-specific factors, particularly psychiatric comorbidities, play a crucial role in treatment continuation. The evidence suggests that medication response and adherence patterns vary significantly across different patient subgroups, emphasizing the importance of personalized treatment approaches in adult ADHD management.

Therefore, we label our patient groups as MA vs. MNA but keep our interpretation that on a biological level medication adherence can reflect both neurobiological factors related to individual patient factors as well as direct treatment effects. Further understanding of the effects of stimulants on an individual level is crucial to interpreting the differences between the MA and the MNA group. It is possible that in the MNA group, higher connectivity reflects a maladaptive response to medication withdrawal, leading to symptom worsening.

Several studies have identified increased neural activation or connectivity patterns in individuals with ADHD that may represent maladaptive responses or compensatory mechanisms. Mattfeld et al. found increased DMN connectivity in ADHD individuals, which correlated with worse clinical outcomes at a one year follow-up, potentially reflecting a failure to suppress DMN activity during task engagement^60^. Shaw et al. observed delayed maturation of cortical thickness in children with ADHD, particularly in the motor cortex, suggesting that increased and prolonged motor cortex activation might be a maladaptive compensatory response to this delay^61^. Additionally, Schulz et al. reported increased frontal-parietal activation during working memory tasks in adolescents with ADHD, which was interpreted as a potentially maladaptive compensatory mechanism for underlying working memory deficits^62^. These findings collectively suggest that stronger activation or increased connectivity in certain brain regions may represent maladaptive responses in ADHD, reflecting compensatory mechanisms or failures in proper neural regulation.

We were able to gain further biological validation for our results through a spatial correlation analyses of MRI data with positron emission tomography derived receptor maps in the NAc. The analyses showed a positive linear correlation between the FC patterns and the expression of the dopamine transporter DAT, D1 and D2 receptors in these areas. Which in turn strengthens the link between the connectivity patterns and underlying dopaminergic neurotransmission in ADHD, aligning with research highlighting dopamine’s role in ADHD pathophysiology^37,63,64^. This correlation reflects the potential of FC measures as a predictive bio-marker, as they may very well reflect the underlying dopaminergic function and thus treatment response as already demonstrated by the divergent outcomes observed in the MA and MNA groups. Furthermore, dopamine modulation in the observed connectivity patterns between the NAc, the DMN and the SN, potentially reflects the differential involvement of direct and indirect basal ganglia pathways^65,66^, which might explain how dopamine modulation affects these large-scale networks in ADHD further underlying neurobiological processes of our FC observations. Our findings align with the recent work by Kaminski et al.^16^ who demonstrated changes in striatal connectivity networks following stimulant exposure, and complement findings from Parlatini et al.^17^ showing how cortical alterations may predict methylphenidate response. These studies, together with our results, show a growing body of evidence supporting the potential use of neuroimaging biomarkers in ADHD treatment prediction^18^.

Some limitations need to be addressed: While we describe NAc connectivity patterns, we are aware that resting state fMRI in our technical setting might not prove optimal to reliably delineate different brain nuclei. In addition, future studies might use more sophisticated surface-based analysis and a more fine-grained analysis of the anterior–posterior shift in the insula’s functional subdivision^67^. Furthermore, resting state fMRI is not directly related to behavioral outcomes. Future research should supplement our findings with task-based behavioral paradigms related to ADHD pathophysiology, such as reward anticipation.

While our sample size (n = 54) may be considered small, it is important to contextualize this within the field of fMRI research, where smaller samples are not uncommon due to the resource-intensive nature of neuroimaging studies. Despite this limitation, we believe our findings remain valuable and robust for several reasons. Using a standard methodological approach, including standard preprocessing techniques and motion correction, have helped ensure high-quality data. FC analyses showed network alterations in NAc FC in our subjects, matching expectation of FC pattern changes in the reward system of ADHD patients. Moreover, our use of multiple comparisons correction methods (e.g., cluster-based thresholding) helps mitigate concerns about false positives. Nonetheless, we acknowledge the limitations inherent in a smaller sample size and emphasize the need for replication in larger cohorts. While our statistical approach helps control for multiple comparisons, small sample sizes can still lead to inflated false positive rates and reduced reliability^68,69^. Future studies should aim to extend these findings with increased statistical power, which may reveal additional subtle effects and allow for more nuanced analyses of individual differences.

Future studies should also include children and adolescents to observe the development trajectory of the reward system FC to the DMN and SN.

In summary, our study found that FC patterns between the reward system and the DMN and SN correlate with the clinical course of ADHD symptoms and the response to medication. Further studying of the reward system and its role in mediating between both networks is of importance, especially in ADHD patients. FC of the reward system to DMN and SN has the potential to be used as a biological marker of response to stimulants-medication and clinical prognosis of ADHD. In conclusion, the correlation between higher NAc-connectivity and divergent treatment outcomes in ADHD patients after three years is a complex phenomenon. The interplay of clinical severity, treatment mechanisms, neuroplasticity, individual variability, and the length of follow-up may all contribute to these findings. Further research, possibly with extended follow-up periods, randomization, and more comprehensive assessments, is needed to better understand the underlying reward system mechanisms and implications for clinical practice.

## Supplementary Information

Supplementary Information 1.

Supplementary Information 2.

## Data Availability

The data sets analysed during the current study are not available publicly due to governing german data protection laws but are available from the corresponding author on reasonable request.
