# Supplementary figures and images for: Functional connectivity of the nucleus accumbens predicts clinical course in medication adherent and non-adherent adult ADHD
Source: Sci Rep. 2025 Jun 4;15:19663. doi: 10.1038/s41598-025-96780-3 (PMC12137675; doi:10.1038/s41598-025-96780-3)

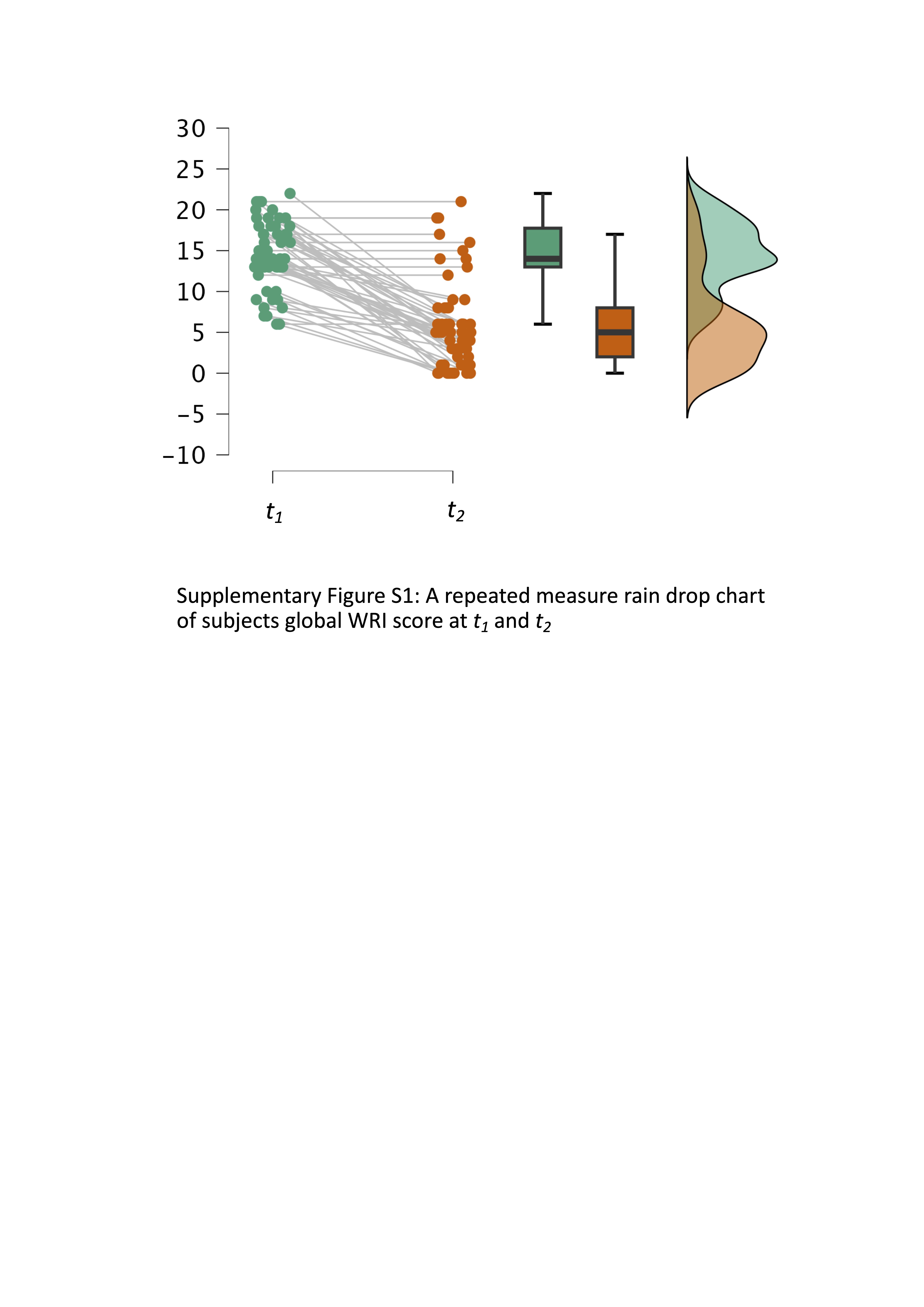

Supplement: Supplementary file 1 — Supplementary Information 1. [file 41598_2025_96780_MOESM1_ESM.jpg]

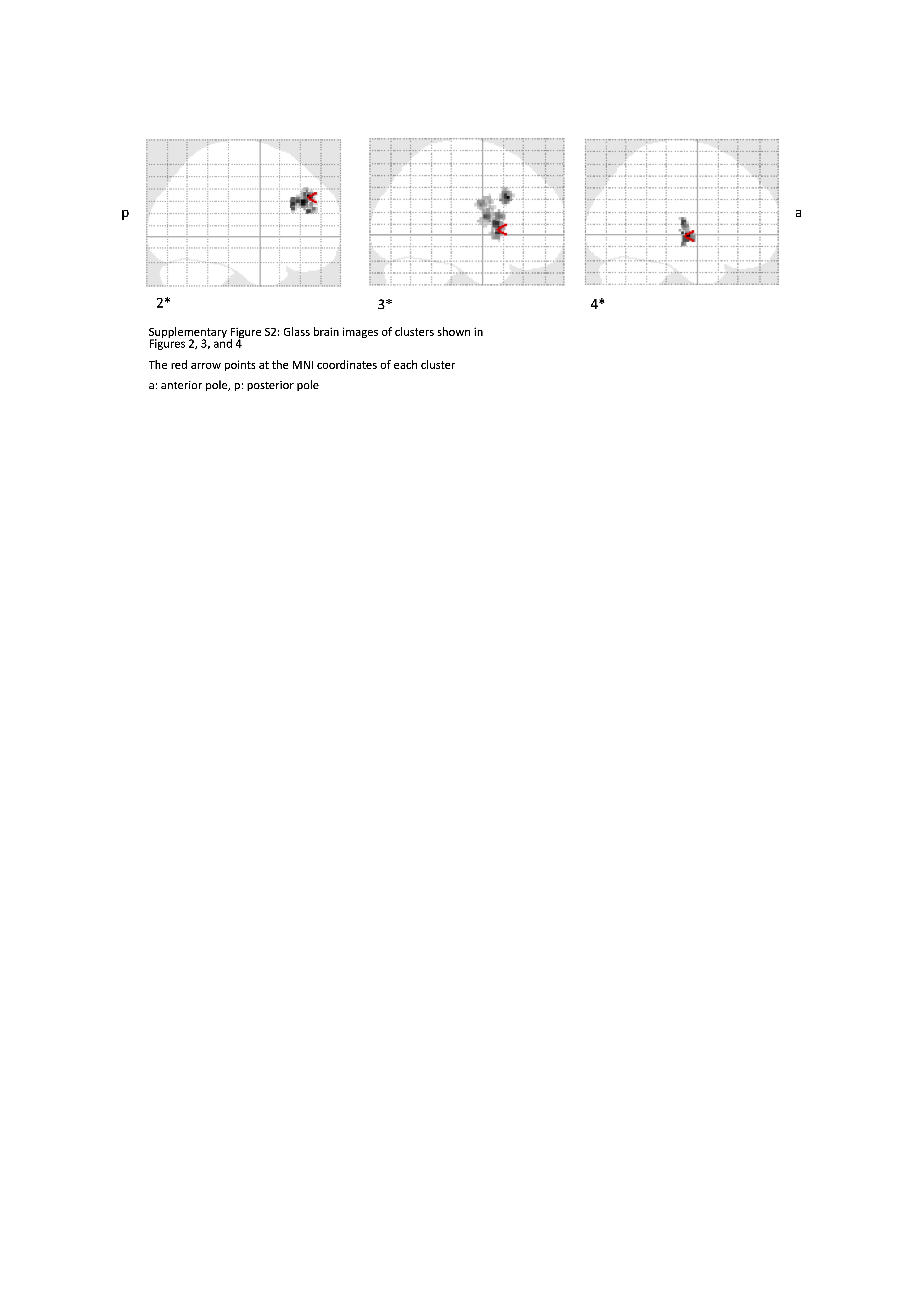

Supplement: Supplementary file 2 — Supplementary Information 2. [file 41598_2025_96780_MOESM2_ESM.jpg]
